# Measurements, Predictions, and Control in Microgrids and Power Electronic Systems

**DOI:** 10.3390/s23084038

**Published:** 2023-04-17

**Authors:** Carlos R. Baier, Jesus C. Hernández, Patrick Wheeler

**Affiliations:** 1 Department of Electrical Engineering, Universidad de Talca, Camino Los Niches Km. 1, Curicó 3344158, Chile; 2 Department of Electrical Engineering, University of Jaén, 23071 Jaén, Spain; jcasa@ujaen.es; 3 Department of Electrical and Electronic Engineering, Faculty of Engineering, University of Nottingham, Nottingham NG7 2RD, UK; pat.wheeler@nottingham.ac.uk

## 1. Overview

The systems used to distribute electricity are currently undergoing a series of changes that are aiding in the development of smart grids [1]. Microgrids and the power electronics involved through grid-connected converters are crucial to achieving this development since they allow for greater flexibility and efficiency in energy management. Furthermore, the fragmentation of distribution systems into multiple controllable subparts improves the global reliability of electrical networks [2]. Fully automated microgrids can operate when connected to main power networks or isolated from them in case of a failure affecting the master grid. However, managing each of the resulting parts and coordinating these parts radically increases the need for measurements, predictions, and control. Smart grids and microgrids started to be seriously studied in the first decade of the 21st century. The reasons for their study mainly point to the dissatisfaction with the operation of traditional electrical networks at the end of the 20th century [3]. The major blackouts of 1965 and 1977 in New York, the one in 1999 in Brazil, and the major blackout in the Northwest of the United States in 2003 only served to demonstrate the limitations and vulnerabilities of traditional electrical systems, reaffirming the need for greater flexibility, automation, and control in the management of energy. The research and development that can still be carried out on these topics are relevant since both microgrids and power electronics help reduce dependence on centralized energy sources and greenhouse gas emissions. The Special Issue “Measurements, Predictions, and Control in Microgrids and Power Electronic System” has aimed to collect new knowledge related to obtaining electrical variables—voltages, currents and/or powers—in real time using new forms of metering/sensing, estimation, or prediction. In particular, recent developments aimed at control applications in systems, such as grid-connected inverters or micro-grids, have been sought after and promoted.

## 2. A Brief Review of the Contributions in This Special Issue

One prevalent issue that affects microgrids, particularly when operating in isolated mode, is the absence of generation-related inertia. Conventional power systems typically have synchronous generators that provide this inertia, allowing them to maintain their rotation speed and frequency, even when there are sudden changes in demand. In contrast, microgrids that rely more on renewable energy sources, such as photovoltaic PV panels, and fewer synchronous generators experience a marked reduction in generation-related inertia. This reduction can cause voltage and frequency fluctuations during load changes or faults. However, these deviations can usually be addressed before they cause significant harm. The paper [4] analyzes the stability of the control system of a back-to-back converter associated with a microgrid with large frequency variation margins. The proposed dynamic decoupling strategies for the controlled variables of the system in Contribution 1 allow it to operate correctly in wide frequency ranges in microgrids. In the same context of microgrids, load surges or significant changes in the generation of renewable systems, such as shading in PV panels, can cause abrupt variations in the control references of the associated converters. Within the same issue, article [5] demonstrates that emerging converters, such as quasi-Z inverters, can use a hybrid control strategy to improve their dynamic response against these abrupt current or power changes in the control references. The proposed scheme in [5] involves a predictive control that only operates during transients, while a linear control strategy operates during stationary states. This way, the converter can maintain better performance, especially during stationary conditions.

The high demand for microgrid requirements and applications has created new prospects, particularly for emerging converters. In addition to the quasi-Z inverters discussed in [5], a new differential inverter is presented in [6]. Differential inverters use various modules of DC–DC converters to obtain alternating currents, taking advantage of the DC voltage differences between the modules. The paper [6] presents a new type of inverter based on Flyback modules operating as a three-phase differential inverter. The proposal considers the mathematical modeling of the system, as well as the modulation and control necessary for its correct operation. Furthermore, [6] demonstrates the feasibility of injecting three-phase power into a microgrid through a practical prototype.

Microgrids benefit greatly from using AC–DC and DC–DC converters, particularly when incorporating energy storage systems (ESS). These ESSs provide a means to address the absence of inertia in the networks, with many of them running on DC power. To manage power in these ESSs, either DC–DC converters or AC–DC converters are required. However, the control of these systems is often complicated, mainly because they are non-linear systems with more than two dynamic variables that need regulation. To reduce the number of sensors in the control of these systems, variable estimation and sensorless control strategies can be employed. Generally, model predictive control (MPC) helps reduce the need for sensors, as predictive models allow for estimating variables required in real-time, where a modular rectification system and a finite-control-set MPC are presented to control the same system. This is demonstrated in [7]

Contributions [8,9] of this Special Issue provide innovative approaches for generalizing sensorless control strategies for different types of DC–DC converters [8] and for rectifying AC–DC using DC–DC converters [9]. In the same context of DC–DC converters, it is well-known that these converters can operate in continuous and discontinuous modes when they operate with synchronous commutation. Successfully operating in the discontinuous mode, without compromising the quality of the electrical energy, can be essential to reducing losses in these systems. Contribution [10] of the Special Issue carries out a performance evaluation of both modes of operation in SEPIC, Cuk, and Zeta converters.

It is essential to highlight that DC microgrids have gained much attention, particularly bipolar DC grids. Because they have a neutral conductor, these grids have the advantage of reducing return currents through the existing neutral. It is easier to detect and protect the system in the event of a fault when compared to unipolar DC grids. The bipolar DC grid concept is more efficient, flexible, and of higher quality than conventional unipolar networks [11]. The paper [12] addresses the problem of constant power flows in a radial bipolar DC grid with multiple unipolar and bipolar loads and proposes a solution. Analyses, including loss calculations, were performed considering both the presence and absence of a ground connection to the neutral in different standard networks, such as the IEEE 85-bus system.

The paper [13] proposes a methodology to minimize the annual cost of energy purchasing and investment in PV sources for a 20-year planning period. The methodology uses a mixed-integer, nonlinear programming model and a master–slave approach with a discrete-continuous version of the Vortex Search Algorithm. The numerical results show the efficiency and effectiveness of the proposed approach for solving the location and dimensioning problem of PV systems in distribution networks. The proposed methodology is independent of the number of nodes in the network, and the solution quality is of greater importance than total processing times.

Finally, this Special Issue includes a review of micro-inverters. In [14], the increasing importance of renewable energy sources and PV modules for electricity generation is discussed. It highlights the use of micro-inverters as a promising solution due to their high efficiency and flexibility. The article reviews different control strategies for micro-inverters, particularly focusing on problems such as partial shading in PV modules. However, it is noted that there has been little research on stability and robustness analysis of these strategies. Contribution [14] also shows that advancements in semiconductor technology and cost reduction have led to increased studies on micro-inverters. The article emphasizes that the design of the control strategy, as well as the selection and design of electronic components, is essential to improve the reliability and functionality of micro-inverters.
